# Diagnostic potential of blood-based biomarkers in multiple sclerosis

**DOI:** 10.3389/fneur.2024.1425046

**Published:** 2024-12-31

**Authors:** Signe Holm Nielsen, Morten Karsdal, Bruna Manoel, Anne-Christine Bay-Jensen, Kim Henriksen

**Affiliations:** Nordic Bioscience, Herlev, Denmark

**Keywords:** blood-based biomarkers, multiple sclerosis, diagnosis, proteins, fragments

## Abstract

Multiple sclerosis (MS) is a chronic inflammatory disease affecting the central nervous system (CNS). There is a significant delay in diagnosing MS as the symptoms and tests overlap with other diseases. Blood-based biomarkers, which quantify fragments of proteins involved in MS pathophysiology, have the potential as diagnostic biomarkers. In this study, we evaluated biomarkers by immunoassays, of tissue destruction, reflected by biglycan degraded by matrix metalloproteinases (MMPs) (BGM), cathepsin S-degraded nidogen (NIC), and MMP-degraded secreted protein acidic and rich in cysteine (SPARC-M) in healthy donors and patients diagnosed with MS. The biomarkers were able to separate the two groups with an AUC = 0.710, AUC = 0.765, and AUC = 0.875, respectively. These pathologically released protein fragments could potentially be used as biomarkers in clinical management providing a specific protein fingerprint.

## Introduction

Multiple sclerosis (MS) is a chronic inflammatory disease affecting myelin sheaths and axons in the central nervous system (CNS) (1). It presents significant inter- and intraindividual heterogeneity regarding the radiological and histopathological changes, clinical presentation, progression, and therapy response. MS is diagnosed using the McDonald’s guidelines (2), where clinical and laboratory evaluations and magnetic resonance imaging (MRI) are performed. Nevertheless, 40% of MS patients have a significant delay in their diagnosis as the diagnostic tests are based on several parameters and share symptom overlap with other diseases such as stroke and migraines (3). Hence, there is a need for biomarkers that precisely and accurately quantify disease activity and may be used as single tools, or in combination with MRI and clinical characteristics (4, 5). Pathologically, the hallmarks of MS are blood–brain barrier (BBB) disruption, infiltration of inflammatory cells, demyelination, axonal destruction, and focal sclerotic plaque formation affecting white matter and, eventually, gray matter (6).

The extracellular matrix (ECM) makes up 20% of the normal brain and controls the progression of MS lesions. In the CNS, cells such as endothelial cells, astrocytes, neurons, and microglia can synthesize and secrete ECM proteins (7). The ECM is involved in the migration, maturation, differentiation, and survival of neurons and maintaining tissue structures. Previous studies have shown how proteases, such as matrix metalloproteinases (MMPs), and ECM components play a role in lesion pathogenesis and CNS dysfunction (8). The ECM proteins biglycan, nidogen, and secreted protein acidic and rich in cysteine (SPARC) have previously been associated with MS lesions. Biglycan and nidogen are found in active and inactive human MS lesions, while SPARC has been found in cerebrospinal fluid in MS patients by proteomics (7, 9).

We hypothesized that biomarkers of extracellular matrix remodeling, quantified by MMP-degraded biglycan, BGM, cathepsin S-degraded nidogen, NIC, and MMP-degraded SPARC (SPARC-M) could be diagnostic biomarkers in patients with MS.

## Methods

Serum samples from patients with MS (*n* = 23) and healthy donors (*n* = 18) were obtained from ProteoGenex (Culver City, CA, United States). Of the MS patients, 14 are diagnosed with primary progressive MS (PPMS), while 9 are diagnosed with relapsing–remitting MS (RRMS). Blood samples were collected according to predefined standard operating procedures by ProteoGenex, and serum was stored at -80°C until biomarker analysis. Samples were collected after informed consent and approval by the local ethical committee in compliance with the Helsinki Declaration of 1975. We examined the immunoassays BGM (10), quantifying MMP-degraded of biglycan, NIC (11), quantifying cathepsin S-degraded nidogen, and SPARC-M (12), quantifying MMP-mediated SPARC (Nordic Bioscience, Herlev, Denmark). All assays are validated for quantification in human serum, and the inter-assay and intra-assay coefficients of variation are <15 and <10%, respectively.

## Results

**Figure 1:**
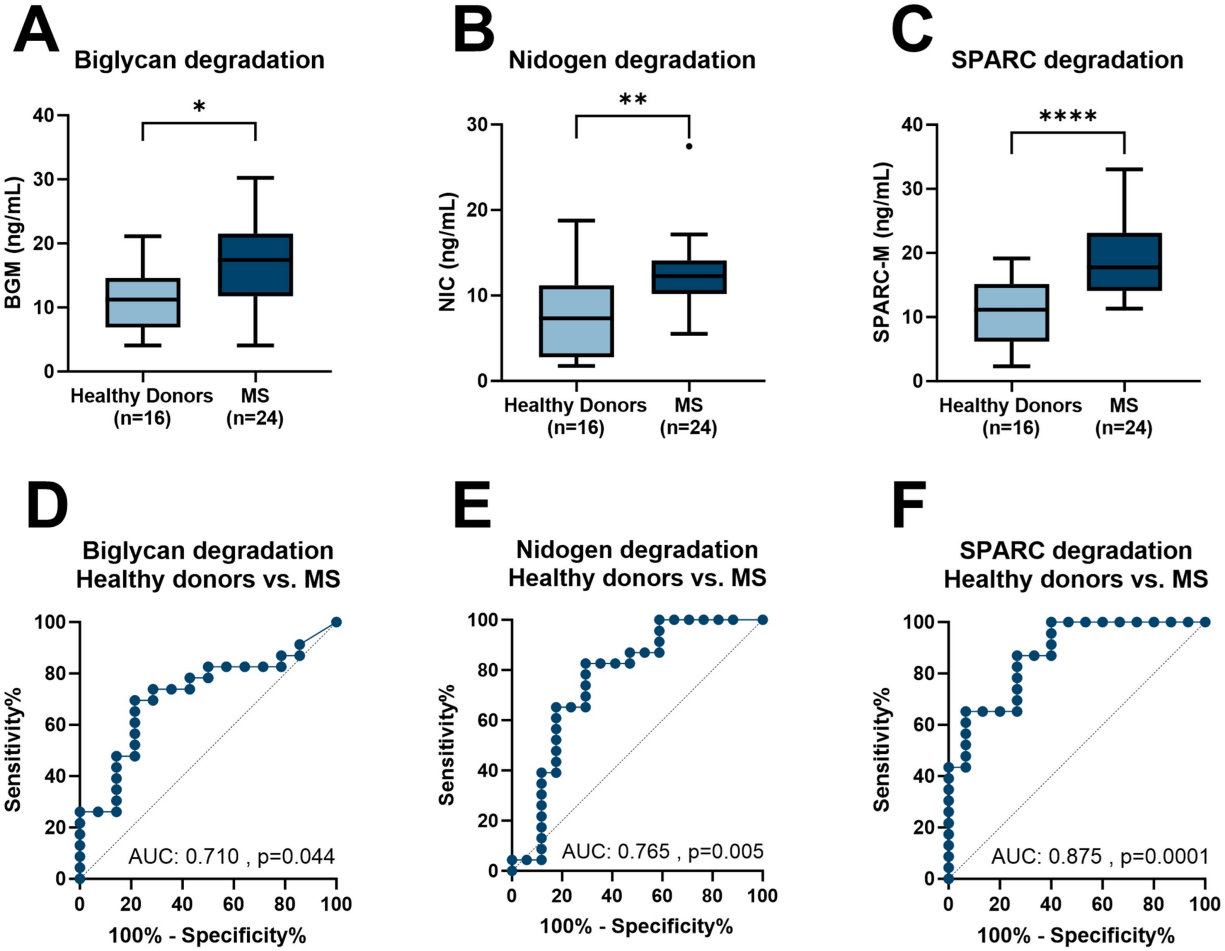
Levels of BGM, NIC, and SPARC-M in serum from healthy donors and patients with MS. **(A)** BGM levels in healthy donors (*n* = 18) and MS (*n* = 24), **(B)** NIC levels in healthy donors (*n* = 18) and MS (*n* = 24), **(C)** SPARC-M levels in healthy donors (*n* = 18) and MS (*n* = 24), **(D)** Receiver operating characteristics (ROC) curve analysis, evaluating the ability of BGM to discriminate between healthy donor’s patients with MS, **(E)** ROC curve analysis, evaluating the ability of NIC to discriminate between healthy donor’s patients with MS and **(F)** ROC curve analysis, evaluating the ability of SPARC-M to discriminate between healthy donor’s patients with MS. Data were analyzed using a Mann–Whitney test, or a ROC curve analysis. Data are presented as Tukey Box Plots. Significance levels: *p < 0.05, **p < 0.01, ****p < 0.0001.

Out of 23 patients with MS (8 men and 15 women), the mean age was 35.7 years (SD = 3.7), and the 18 healthy donors (9 men and 9 women) had a mean age of 35.8 (SD = 3.8) years as reference (Table 1). All three biomarkers, namely, BGM, NIC, and SPARC-M, were significantly elevated in patients with MS compared to healthy donors (*p* = 0.028, *p* = 0.004, and *p* < 0.0001, respectively, Figures 1A–C). In addition, we investigated the diagnostic accuracy using the area under the ROC curve (AUROC) of BGM for patients diagnosed with MS compared to healthy donors of 0.717 (95%CI: 0.547–0.888, *p* = 0.028, Figure 1D), NIC for patients diagnosed with MS compared to healthy donors of 0.765 (95%CI: 0.601–0.928, *p* = 0.005, Figure 1E), SPARC-M for patients diagnosed with MS compared to healthy donors of 0.875 (95%CI: 0.764–0.987, *p* < 0.0001, Figure 1F). There were no significant differences in the biomarker levels of BGM, NIC, or SPARC-M between patients diagnosed with either PPMS or RRMS.

**Table 1:** Patient demographics.

	Healthy donors (*n* = 18)	Multiple Sclerosis (*n* = 23)	*p*-value
Age, mean (SD)	35.8 (3.8)	35.7 (3.7)	0.908
Sex, Male (%)	9 (50%)	8 (34.78%)	
BMI	25.6 (2.96)	24.8 (3.25)	0.426
Caucasian (%)	25 (100%)	24 (100%)	1.000
Smoking, current smokers (%)	0 (0%)	2 (8.7%)	
Years since diagnosis (SD)	NA	1.3 (2.22)	

Categorical variables are written as numbers (percentage), while continuous variables are mean (standard deviation). BMI, body mass index.

## Discussion

In this study, we evaluated BGM, NIC, and SPARC-M levels in a cohort of healthy donors and patients with MS to investigate their potential as diagnostic biomarkers. There is a need for blood-based biomarkers in MS, to help understand the pathogenesis, monitor disease progression, and tailor treatment strategies. Based on these results, SPARC-M had the best diagnostic potential, confirming findings in literature where SPARC was modulated in cerebrospinal fluid from MS patients (9). In addition to its presence in cerebrospinal fluid, it has been associated with TNF-*α*-induced BBB dysfunction, indirectly related to the pathophysiology of MS. In addition to this, BGM and NIC are also upregulated in serum from MS patients, confirming the presence of these proteins together with infiltrating immune cells in MS lesions (7).

One limitation of this study is the specificity of the blood-based biomarkers quantified in this cohort of patients. ECM proteins are distributed throughout the body and are not brain lesion-specific. However, as in other diseases, there could be value in determining a combination of non-specific individual markers, which in combination increase the specificity for MS.

There is a need for blood-based biomarkers in MS to help understand the pathogenesis, monitor disease progression, and tailor treatment strategies. Utilizing ECM biomarkers which are involved in the pathogenesis of BBB dysfunction, together with infiltrating immune cells in the lesions, may describe a part of the pathophysiology and help elucidate the complexity of MS disease. In summary, this exploratory study showed that blood-based biomarkers targeting the ECM are associated with MS pathology and may potentially be used as biomarkers in clinical management.

## Data Availability

The raw data supporting the conclusion of this article will be made available by the authors, without undue reservation.
